# Institutional Notes and News

**Published:** 1910-04-23

**Authors:** 


### Institutional Notes and News.

A festival dinner was held last week at the Mansion House in aid of the Miller General Hospital and Royal Kent Dispensary. The Lord Mayor, who presided, reminded those present that that part of the hospital's income which was reliable only amounted to £450. Mr. Justice Darling also spoke, pointing out that South-East London meant a population of 510,000, and in order that the Miller Hospital might cope with the work that brought to its doors £5,000 a year was needed. The hospital's Treasurer, Mr. Benn, said that last year expenditure exceeded income by £1,000, and that more beds were wanted. At present there are only twenty-five, which, the Bishop of Southwark said, were 200 too few. The sum of £1,205 was announced at the dinner as received.

**Miller Hospital Dinner.**

Plenty of interesting business was transacted at the recent annual meeting of the Royal Boscombe and West Hants Hospital, at Bournemouth. Mr. Walter Child Clark, the vice-president, who took the chair in the absence of the president, Sir Gilbert Wills, raised much laughter by comparing the efficiency of the hospital inside, which his experience as sanatorium vice-president and hospital chairman at Liverpool gave him power to judge, with its unfortunate appearance without. "If only," Mr. Clark said, "some rich men of the county would make the exterior something like what it appeared to be in the illustrations," it would be a great improvement. The total debt and overdraft on the maintenance account unfortunately adds up to £4,250. By way of setting an example to subscribers generally Mr. Clark announced that he and his wife would treble their subscriptions. A hundred-thousand shilling fund is also being started. The constant enthusiasm and generosity of Mr. Clark to the hospital were publicly acknowledged by the Rev. E. J. Kennedy, who proposed a resolution thanking him for one of his latest gifts, the new children's ward. This ward, it was resolved by the governors, should be known as the "Walter Child Clark Ward" to all posterity.

**Royal Boscombe and West Hants Hospital.**

Mr. G. Whitcombe, a Governor of the Gloucestershire Royal Infirmary and Eye Institution, congratulated the hospital at the recent annual meeting on the most satisfactory report he had heard for many years. Colonel Curtis Hayward, who presided, remarked that the year had been a very busy one, but memorable from the fact that the King, on his visit to Gloucester, had granted the hospital the style of "Royal." A record number of in-patients had been admitted, 1,591 in number, giving the hospital an average of 90 occupied beds. This average is one fewer than last year, in spite of the increased number of admissions, because the average length of stay in the wards has been reduced to 20.6 days. The hospital's income was £7,024 and its expenditure £7,744, leaving a debt which amounted to £720. This, however, is, Colonel Hayward pointed out, a matter of congratulation by comparison, for if the figures are analysed and placed beside those of preceding years, it appears that the income has increased by £193 and the expenses have been reduced by £240. At the last meeting the committee recorded a debt of over £1,000, but by Mrs. Calvert's Gloucestershire Royal Infirmary League (modelled, we may note, on the League of Mercy) many non-subscribers have been gathered in. Mr. James P[illegible]uton, who was Mayor at the time of the King's visit, and Mr. Russell Rea were thanked at the meeting for their action in connection with the Royal acknowledgment of the hospital.

**Royal Infirmary, Gloucester.**

We have received the report of the Royal Alexandra Infirmary, Paisley, N.B. The number of patients treated in the Infirmary is given as 1,328. The accounts are interesting, because they contain one item that appears to be new in the accounts of this particular hospital. This item is the sum received from paying pupils as a fee for their training as nurses in the Infirmary. This experiment produced £21 last year, and the report hopes that "it may prove successful alike in the interests of the institution and the pupil probationers." The Infirmary's income was £6,989, its expenditure £7,424, leaving it £381 in debt at the end of the year. Previous debts have been accumulating, however, and the net sum now owed is £864. Mr. Peter Coats's career of generosity to the infirmary still knows no abatement. The Nursing Home which he added to extensively in 1908, the redecorations which he carried out in the original building in 1909, have now been succeeded by his gift of a site in Stock Street. This has been cleared of houses, prepared for two tennis courts, surrounded by a wall, and, as if not content with this, Mr. Coats has secured to the Infirmary the first refusal of the bowling green adjoining, in case of any future plan for building on, or selling it. The endowment fund continues to grow owing to the principle, which is sincerely followed, of placing to its credit all legacies that are not ear-marked for anything else.

**Alexandra Infirmary, Paisley.**

Progress is being made with the scheme to provide Glasgow and the West of Scotland with a model children's hospital. The state of affairs, we may remind our readers, at the beginning of the year was as follows: The directors of the present Royal Hospital for Sick Children, Glasgow, had approved of a scheme, the final object of which was to build a hospital on the pavilion system for 300 cots, at a cost of £120,000. By this scheme some £100,000 has been raised, and a splendid ten-acre site purchased on the top of York Hill. The choice of a site was the subject of some controversy, but York Hill presents the solid advantages of a healthy situation, a central position, and closeness to the University. This last will make the hospital available as a medical school for the study of children's diseases, from which both hospital and University will benefit. In this connection we cannot resist quoting some remarks by Dr. Barclay Ness: "The good work which the present institution is doing," he said, "is not only in the healing of children, but in the educating of parents. For the most common cause of illness in the case of children attending the hospital is the ignorance of the mothers, and it is only in so far as we succeed in teaching the mothers that lasting benefit can be conferred upon the children." The extended hospital will therefore be a school in more than one sense, and a school very much needed. Great care has been taken with the plans, under the expert advice of Dr. Donald Mackintosh, who, with Mr. John James Burnet, the architect, has visited the most up-to-date children's hospitals in America and Germany, in search of ideas and to compare results. These results have been brought to Glasgow and embodied in the complete plans. The building, we believe, is now begun.

**Glasgow New Children's Hospital.**

